# Non-infective Unilateral Panuveitis: Topical Steroids and Posterior Vitrectomy as a Cheap and Safe Alternative to Modern Treatment Modalities

**DOI:** 10.7759/cureus.5587

**Published:** 2019-09-06

**Authors:** Zauraiz Anjum, Zemal Tariq

**Affiliations:** 1 Internal Medicine, Fatima Jinnah Medical University, Lahore, PAK; 2 Internal Medicine, Gujranwala Medical College, Gujranwala, PAK

**Keywords:** non-infective, unilateral, panuveitis, treatment, posterior vitrectomy, topical steroids, steroid responder

## Abstract

Uvea of the eye is a term that includes the iris, choroid, and ciliary body. Inflammation of all layers of the uvea is called panuveitis. Panuveitis can even spread to involve the optic nerve, retina, vitreous humor, or lens. This process can lead to redness and pain in the eye, blurring of vision, and even blindness. The condition is usually treated with topical steroids, but it becomes difficult in steroid-responders. Here, we present the case of a rare non-infective unilateral case of panuveitis that was successfully treated in an unorthodox manner.

## Introduction

The iris, choroid, and ciliary body make up the uvea of the eye [1-6]. These structures can get inflamed, and the inflammation presents as floaters, redness, and pain in the eye [1-6]. The inflammation can further involve the lens, vitreous, retina, and optic nerve, leading to blurring of vision initially and to loss of vision eventually [1-6]. The condition has traditionally been treated with steroids. Oral steroids have many side effects, and steroids, as a class, are especially dangerous for a subset of the population called ‘steroid-responders’ who readily develop glaucoma when treated with steroids [7]. Our team had to deal with such a case. We treated the patient in a way that ensured proper delivery with limited side-effects.

## Case presentation

History and examination

An 18-year-old male presented to the outpatient ophthalmology department with a complaint of worsening of blurring of vision in his left eye. On further inquiry, the patient revealed that he was 10 years' old when he first started seeing floaters in his left eye. He did not seek medical attention at the time. The condition worsened gradually to blurred vision, especially in times of stress. He reported that his mother has sarcoidosis. He denied any history of trauma to the eye, any joint pain, or exposure to a patient with tuberculosis.

His past medical, social, or sexual history was not significant. The ophthalmologic examination, including the slit-lamp examination, revealed left-sided cataract and keratic precipitates. His optic disc showed signs of active inflammation. The rest of the physical examination was within normal limits.

Investigations

His complete blood count (CBC), chest X-ray, lumbosacral spinal X-ray, human immunodeficiency syndrome (HIV) by enzyme-linked immunosorbent assay (Elisa), T-spot test, C-reactive protein (CRP), rheumatoid arthritis (RA) factor, and antinuclear antibodies (ANA) and extractable nuclear antigen (ENA) profile were non-significant, and the only abnormality was a slightly raised erythrocyte sedimentation rate (ESR) (22 mm in the first hour by the Westergren method).

Treatment, hospital stay, and results

He was started on oral steroids owing to the involvement of the optic disc. Methylprednisolone at the dose of 60 mg/day was started and gradually tapered over four weeks. As a result of treatment with steroids, the patient developed Cushingoid features. This mode of treatment was also complicated by an episode of Addisonian crisis when the patient did not take his dose during ‘Ramadan’ when he was fasting as a religious duty. The crisis was fortunately promptly managed with injectable steroids and fluids.

Oral steroids were thus switched to topical steroids. The patient turned out to be a steroid-responder and presented to the ophthalmologic emergency with an episode of acute glaucoma. The acute event was managed with topical acetazolamide, and the dose of topical steroids was reduced and topical diclofenac was added. Oral and topical acetazolamide was also added to avoid another episode of glaucoma. The patient was asked to follow up weekly to have his ocular pressure checked.

Three weeks later, a routine ophthalmologic exam revealed macular neovascularization and macular edema. This occurrence presented a unique problem, as the patient could not receive a depot of steroids in his eye for fear of glaucoma. So, the patient was given an intravitreal injection of bevacizumab and prescribed oral methotrexate. The patient found bevacizumab very expensive but agreed to one dose. He also reluctantly took methotrexate (for fear of side effects) for a few weeks and then asked to be taken off of the medicine.

At this time, his cataract had progressed to the point where he asked for an artificial lens. Our team performed phacoemulsification and placed a unifocal lens in his left eye. At the same time, we performed posterior vitrectomy for the unorthodox purpose of allowing the topical medications to reach his retina easily and a canal of Schlemmectomy to counter the ‘glaucoma’ effects of steroids. As a result of this treatment modality, he reported a marked improvement in his vision.

The patient was discharged in a stable condition on topical steroids, topical diclofenac, and an ocular multivitamin. He was asked to follow up weekly initially, then monthly, and three-monthly later on. During his last follow-up (about a year after the procedure), he reported no new symptom, no episode of glaucoma, and improved vision. The slit-lamp examination showed that his inflammation had settled.

## Discussion

Uveitis can be anterior, posterior, intermediate, or panuveitis. Anterior uveitis refers to an inflammatory process occurring in the anterior segment of the eye. This type of uveitis involves inflammation of the iris (iritis) and anterior chamber and anterior vitreous (iridocyclitis). Intermediate uveitis is an inflammatory process that affects mainly the vitreous. Posterior uveitis is an inflammation that involves the retina, choroid, or optic disc primarily [1-6].

Panuveitis refers to the inflammation of all three layers of the eye, which include the iris, choroid, and ciliary body. The inflammation can spread to involve the lens, vitreous, retina, and optic disc as well. The causes of uveitis can be broadly classified as infective, inflammatory, traumatic, or toxic. However, mostly, the cause remains unknown. The infective causes include herpes simplex, syphilis, Lyme disease, toxoplasmosis, HIV, tuberculosis (TB), etc. The inflammatory causes include inflammatory bowel disease, rheumatoid arthritis, systemic lupus erythematosus, sarcoidosis, ankylosing spondylitis, etc. Also, trauma and cigarette smoking can lead to the condition [1-6].

In one of the articles we reviewed, the authors, while working in a university hospital, found that out of the 690 patients with uveitis, 59% were women [8]. The condition was found to be unilateral in more than half of the cases (53%) [8]. The authors found that panuveitis was the most common presentation (52%) vs anterior (30%), posterior (16%), and intermediate (3%) [8]. The cause was found to be infectious in 13% of the patients and non-infectious in 35% [8]. In 52% of the cases, the cause remained unknown [8]. They further found that after 2011, the diagnostic yield had improved [8].

According to another study carried out in Iran, anterior uveitis was found to be the most common (42.9%) vs posterior (21.4%), intermediate (19.3%), and panuveitis (16.31%) [9]. Among the patients in whom a cause was found, the cause was mostly non-infectious while in a large number of patients (43.9%), the cause remained unfound [9].

The signs and symptoms of uveitis can include floaters, redness and pain in the eye, blurring of vision, and photophobia. Limbal injection (conjunctival hyperemia), keratic precipitates and flare, clumps of inflammatory cells, macular edema, and fluid leak from macular neovascularization may be seen on the slit-lamp examination. There may also be posterior synechiae, retinal vasculitis, and inflammatory changes in the retina. The inflammatory process can lead to lens damage and collection of debris in the vitreous, leading to further deterioration of vision [1-6].

Diagnosis is usually made based on history and physical examination. Ophthalmoscopy, fundoscopy, and the slit-lamp examination provide useful evidence in support of the diagnosis [1-6].

The mainstay of treatment has traditionally been steroids and cycloplegic-mydriatic drugs for ‘resting’ the eye. The steroids can be given orally but are preferred topically or by periocular or intraocular injection. This modality is very dangerous in the subset of population called ‘steroid-responders’ who have a heightened tendency to develop acute glaucoma in response to steroids [7]. Another therapy that has been tried is with immunosuppressive drugs, e.g., methotrexate or cyclosporin. The immunosuppressants carry a multitude of severe side effects and are thus undesirable to most. Novel therapies include biologics, e.g., adalimumab, laser phototherapy, or transscleral cryotherapy to the peripheral retina. The novel therapies are unfortunately costly, require specialized centers, and are not easily or widely available [1-6].

Our patient, as discussed above, was a case of idiopathic unilateral panuveitis. The diagnosis was supported by typical history and examination, with further information being furnished by the slit-lamp examination. He was a steroid-responder and due to this and other financial, as well as availability restraints, could not be treated by traditional or novel means. He was eventually successfully maintained in a stable condition by a combination of posterior vitrectomy, topical steroids, topical diclofenac, and canal of Schelectomy. His vision was restored to a significant degree by the use of a unifocal lens. The multifocal lens was avoided due to the complicated nature of the case.

Our case report suggests that the mentioned combination of therapies can be used as a cheap and safe alternative to traditional and novel therapies.

## Conclusions

Uveitis is a widespread ophthalmological problem. Although many treatments are well-known, they carry adverse side effects, e.g., steroids, immunosuppressants, etc.; are very expensive, e.g., adalimumab; invasive, e.g., transscleral cryotherapy; or cannot penetrate to the posterior parts of the eye, e.g., topical steroids. Our team used posterior vitrectomy to create a favorable pathway for the topical medications to reach the posterior-most part of the eye, thus avoiding side effects, with minimum expenditure and invasion. More studies are required to find the risk-to-benefit ratio and the long-term effects on mortality, morbidity, and lifestyle with this procedure.
